# Tenovin-6 inhibits proliferation and survival of diffuse large B-cell lymphoma cells by blocking autophagy

**DOI:** 10.18632/oncotarget.14741

**Published:** 2017-01-19

**Authors:** Hongfeng Yuan, Meilan He, Fan Cheng, Rosemary Bai, Suzane Ramos da Silva, Ricardo C.T. Aguiar, Shou-Jiang Gao

**Affiliations:** ^1^ Department of Molecular Microbiology and Immunology, Keck School of Medicine, University of Southern California, Los Angeles, CA, USA; ^2^ Department of Medicine and Cancer Therapy and Research Center, University of Texas Health Science Center at San Antonio, San Antonio, TX, USA; ^3^ South Texas Veterans Health Care System, Audie Murphy VA Hospital, San Antonio, TX, USA

**Keywords:** tenovin-6, diffuse large B-cell lymphoma, autophagy, sirtuins, p53

## Abstract

Diffuse large B-cell lymphoma (DLBCL) is one of the most aggressive non-Hodgkin lymphomas. It is curable but one-third of cases are refractory to therapy or relapse after initial response highlighting the urgent need for developing novel therapeutic approaches. Targeting sirtuins, particularly SIRT1 by genetic approaches or using pharmaceutical inhibitor tenovin-6, has shown promising therapeutic potential in various hematopoietic malignancies. However, it remains unknown whether these approaches are effective for DLBCL. In this study, we have found that tenovin-6 potently inhibits the proliferation and survival of DLBCL cells. Surprisingly, specific knockdown of SIRT1/2/3 has no effect on DLBCL. Mechanistically, tenovin-6 increases the level of microtubule-associated protein 1 light chain 3B (LC3B)-II in a SIRT1/2/3- and p53-independent manner in DLBCL cell lines. Tenovin-6-mediated increase of LC3B-II is through inhibition of classical autophagy pathway. Furthermore, inhibition of the autophagy pathway by using other inhibitors or by knocking down key genes in the pathway impairs cell proliferation and survival of DLBCL cells. These results indicate that targeting the autophagic pathway could be a novel therapeutic strategy for DLBCL and that precaution should be taken to interpret data where tenovin-6 was used as an inhibitor of sirtuins.

## INTRODUCTION

Diffuse large B cell lymphoma (DLBCL), the most common B cell tumor in adults, displays marked clinical heterogeneity. Indeed, first-line immunochemotherapy (R-CHOP-Rituximab, Cyclophosphamide, Doxorubicin Hydrochloride, Oncovin and Prednisone) cures only ~60% of the patients [1]. An emerging consensus in the field is that a better understanding of DLBCL biology may be the most effective way to improve the survival rate of patients who are refractory or have a relapse [2]. To that end, there has been steady progress in elucidating the pathogenesis of DLBCL. In earlier studies, gene expression profiling analyses have defined DLBCL signatures that are associated with a putative cell-of-origin (germinal center and activated B cell, GCB- and ABC-DLBCL) or mechanisms of transformation (consensus cluster classification, CCC) [3, 4], observations that have already guided rational clinical initiatives. More recently, massively parallel sequencing uncovered previous unsuspecting genetic lesions in DLBCL [5], thus suggesting that important elements of DLBCL biology remain to be elucidated creating novel opportunities for tailored therapeutic interventions.

Sirtuins are highly conserved NAD^+^-dependent protein lysine modifying enzymes with deacetylase, ADP-ribosyltransferase and/or deacylase activities. Among this family of proteins, SIRT1 is the most-studied member, and is a key regulator for a wide variety of physiological and pathological processes such as genome stability, metabolism, energy homeostasis, organ development, aging and cancer [6–8]. It has been shown that SIRT1 promotes the survival and/or chemoresistance of various hematopoietic malignancies including chronic myelogenous leukemia [9–11], acute myeloid leukemia [12], acute lymphoblastic leukemia [13], and Kaposi's sarcoma-associated herpesvirus (KSHV)-transformed cells [14]. Targeting SIRT2 can also induce granulocytic differentiation in acute promyelocytic leukemia [15]. Despite these works, it remains unknown whether SIRT1, SIRT2 and other sirtuins have any roles in DLBCL and whether they can be exploited as therapeutic targets.

Tenovin-6, a small molecular compound, has drawn much attention because it can potently activate p53, and directly inhibit the protein deacetylase activity of purified human SIRT1, SIRT2 and SIRT3 [16, 17]. Tenovin-6 has since been used as an inhibitor of sirtuins, especially SIRT1, and has been demonstrated to have a promising anti-neoplastic effect *in vitro* and *in vivo* on various hematopoietic malignancies of both lymphoid and myeloid lineages [9–13, 15, 18, 19]. However, whether tenovin-6 is effective against DLBCL has not been investigated so far.

In this study, we aim to determine whether targeting sirtuins by using genetic approaches or pharmaceutical inhibitor tenovin-6 has any effects on DLBCL. We demonstrated that tenovin-6 could significantly inhibit the proliferation and survival of DLBCL cell lines through SIRT1/2/3-independent inhibition of autophagy.

## RESULTS

### Tenovin-6 inhibits proliferation and survival of DLBCL cells

**Figure 1:**
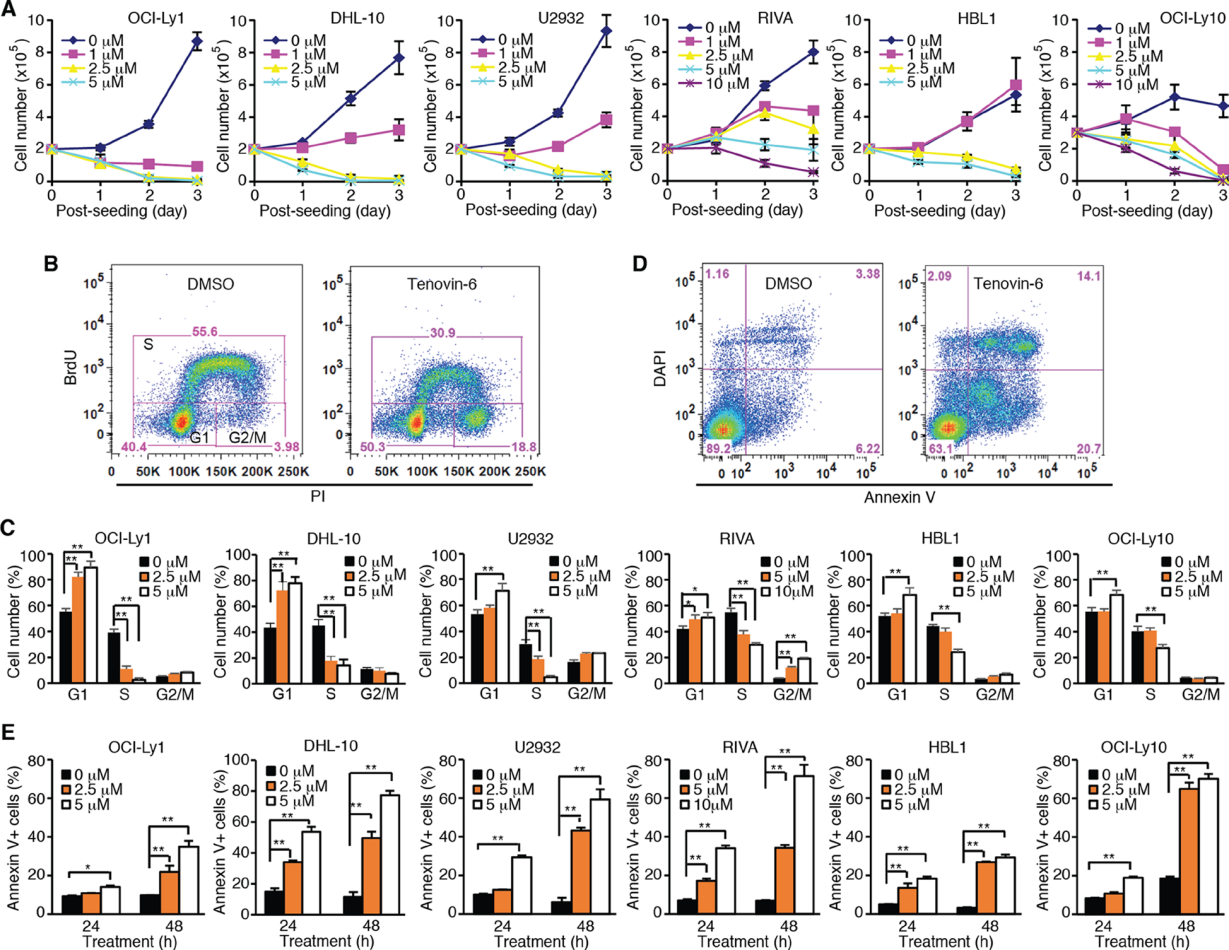
Tenovin-6 inhibits cell proliferation and induces apoptosis of DLBCL cells (**A**) Growth curves of indicated cell lines after treating with indicated doses of tenovin-6. (**B**) Representative profile of BrdU-PI staining in RIVA cells after treatment with 10 μM tenovin-6 for 24 h. (**C**) Percentages of cells at G1, S and G2/M phases of the indicated cell lines after tenovin-6 treatment based on BrdU-PI staining as shown in B. *P < 0.05, **P < 0.01. (**D**) Representative profile of annexin V-DAPI staining in OCI-Ly1 cells after treatment with 5 μM tenovin-6 for 48 h. (**E**) Percentages of annexin V+ cells of the indicated cell lines after tenovin-6 treatment based on annexin V-DAPI staining as shown in D. *P < 0.05, **P < 0.01.

To test whether tenovin-6 had an inhibitory effect on DLBCL, we treated 2 GCB-type DLBCL cell lines OCI-Ly1 and DHL-10, and 4 ABC-type DLBCL cell lines U2932, RIVA, HBL1 and OCI-Ly10 with 0, 1, 2.5, 5 or 10 μM tenovin-6, and counted the viable cells every day for 3 days. Tenovin-6 potently inhibited cell proliferation in a dose- and time-dependent manner in all 6 cell lines (Figure 1A). Examination of cell cycle progression by BrdU (5-bromo-2′-deoxyuridine) and PI (propidium iodide) staining showed that the percentages of cells in G1 phase were significantly increased while the percentages of cells in S phase were significantly decreased in a dose-dependent manner in all 6 cell lines at 24 h post-treatment (Figure 1B and 1C). Furthermore, tenovin-6 induced apoptosis in a dose- and time-dependent manner in all 6 cell lines (Figure 1D and 1E). These results indicated that tenovin-6 potently inhibited cell proliferation and survival of DLBCL cells.

### Knockdown of SIRT1, 2 or 3 in DLBCL cells has no significant effect on cell proliferation and survival

**Figure 2:**
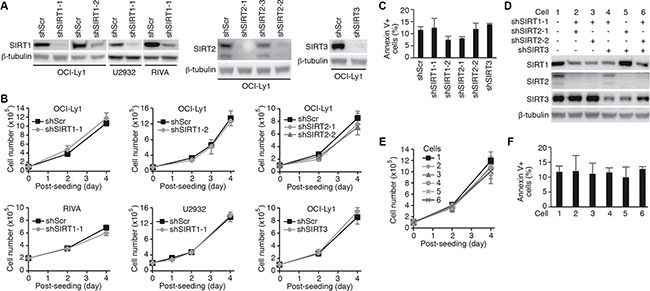
Knockdown of SIRT1, 2 or 3 in DLBCL cells has no effect on cell proliferation and survival (**A**) Knockdown of SIRT1, 2 or 3 in the indicated cell lines examined by Western-blotting. (**B**) Growth curves of the indicated cells following knockdown of SIRT1, 2 or 3. (**C**) Percentages of annexin V+ cells in the indicated cell lines after knockdown of SIRT1, 2 or 3. (**D**) SIRT1, 2 and 3 expression levels in OCI-Ly1 cells following knockdown with different combinations of shRNAs examined by Western-blotting. (**E**) Growth curves of the OCI-Ly1 cells following knockdown with different combinations of shRNAs to SIRT1, 2 or 3. (**F**) Percentages of annexin V+ cells in OCI-Ly1 cells following knockdown with different combinations of shRNAs to SIRT1, 2 or 3.

To determine the roles of sirtuins in DLBCL, we examined the expression of sirtuins in 10 different cell lines (Supplementary Figure 1). All seven members of sirtuins were expressed in all the 10 cell lines examined though the expression levels varied among the cell lines. Since tenovin-6 is thought to be specific to SIRT1, 2 and 3 [17], we performed knockdown of these sirtuins with specific shRNAs. We achieved 90% knockdown of SIRT1 in OCI-Ly1, U2932 and RIVA cells, and SIRT2 or SIRT3 in OCI-Ly1 cells (Figure 2A). However, knockdown of SIRT1, 2 or 3 affected neither the cell proliferation (Figure 2B) nor apoptosis (Figure 2C) of these cells. We then assessed the effects by combining shRNAs to different sirtuins (Figure 2D). Again, we failed to observe any significant effect on cell proliferation (Figure 2E) and apoptosis (Figure 2F). These results indicated that the effect of tenovin-6 was not due to its inhibitory effect on SIRT1, 2 and 3.

### Tenovin-6 consistently increased LC3B-II level in DLBCL cell lines without activating p53

**Figure 3:**
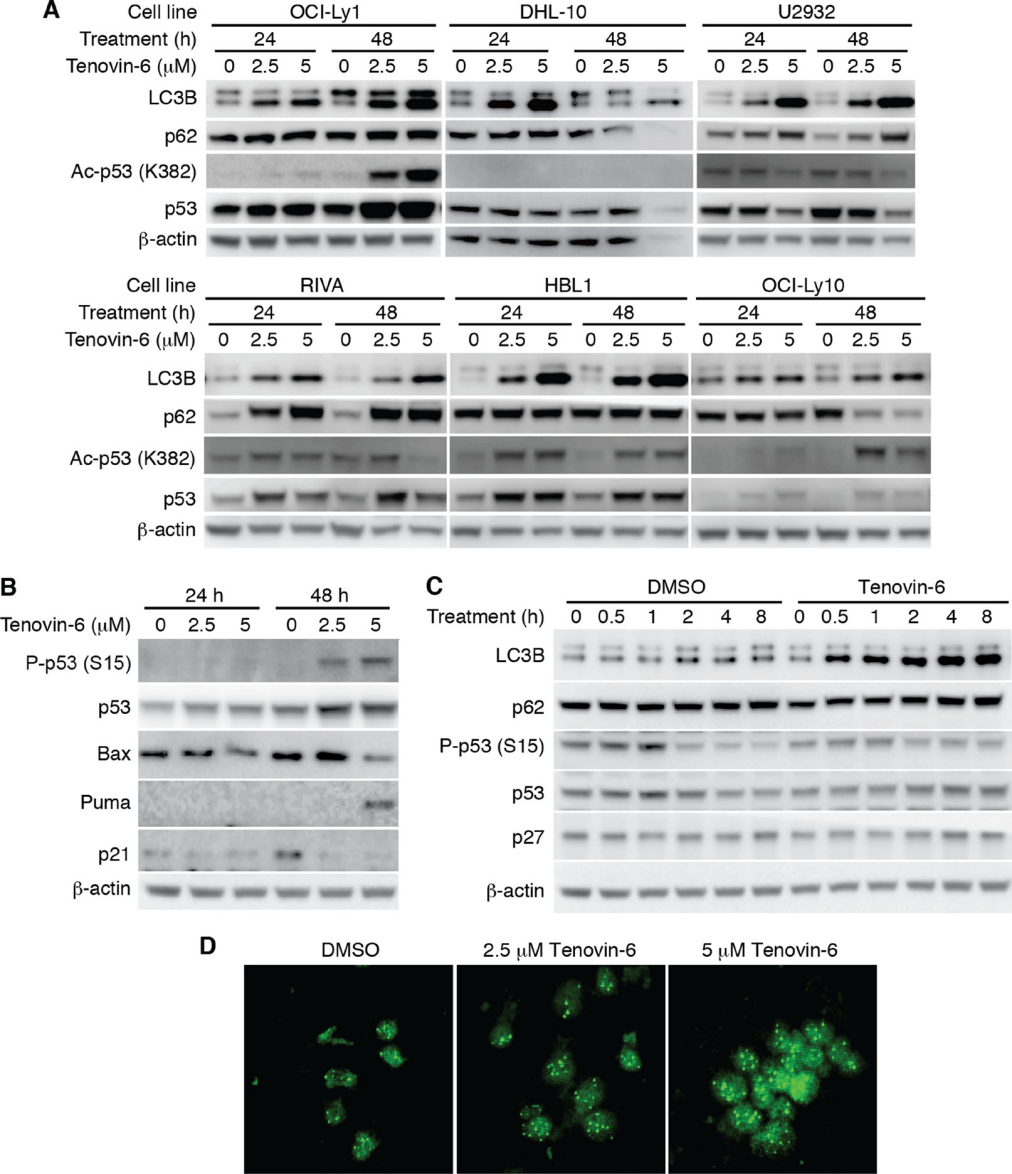
LC3B-II was consistently increased but not p53 activation after tenovin-6 treatment in DLBCL cell lines (**A**) The levels of LC3B, SQSTM1/p62 (p62), acetylated p53 [Ac-p53 (K382)], and p53 in the indicated cell lines after tenovin-6 treatment. (**B**) The levels of phospho-p53 (Ser15) [P-p53 (S15)], p53, Bax, Puma, p21 in OCI-Ly1 cells following tenovin-6 treatment. (**C**) The kinetics of LC3B, SQSTM1/p62, P-p53 (S15), p53 and p27 levels in OCI-Ly1 cells following treatment with 5 μM tenovin-6. (**D**) Immunofluorescence staining of LC3B punta in OCI-Ly1 cells following treatment with 2.5 μM or 5 μM tenovin-6 for 16 h.

To identify the mechanism of tenovin-6 inhibition of DLBCL cells, we examined p53 acetylation as tenovin-6 was initially identified as a p53 activator through inhibition of SIRT1-mediated deacetylation of p53 [17]. We detected increased p53 acetylation in OCI-Ly1, HBL1 and OCI-Ly10 cells but not in DHL-10, U2932 and RIVA cells (Figure 3A). p53 is mutated in OCI-Ly1 [20], DHL-10 [21], U2932 [22, 23], RIVA (also known as RI-1, Broad-Novartis Cancer Cell Line Encyclopedia, https://www.broadinstitute.org/ccle), and HBL1 [22] while it is intact in OCI-Ly10 cells [24]. To further determine whether p53 was activated through other mechanisms following tenovin-6 treatment, we examined p53 phosphorylation and p53 downstream targets Bax, Puma and p21 in OCI-Ly1 cells. While p53 phosphorylation (Ser15) and Puma level increased at 48 h after tenovin-6 treatment, the levels of other p53 downstream targets Bax and p21 did not (Figure 3B). These results indicated that p53 activation was unlikely the mechanism that mediated the observed effect of tenovin-6 on DLBCL cells.

In contrast to the conflicting results of p53 activation, tenovin-6 consistently increased the level of autophagy marker LC3B-II in all the DLBCL cell lines (Figure 3A). In addition, tenovin-6 increased the expression of LC3A-II in U2932, RIVA and HBL1 cells but not in OCI-Ly1 and OCI-Ly10 cells (Supplementary Figure 2). Interestingly, we did not observe any obvious SQSTM1/p62 degradation (Figure 3A and 3C), suggesting that tenovin-6 might inhibit autophagy in these cells. To confirm the increase of LC3B-II, we performed immunofluorescence assay in OCI-Ly1 cells using a LC3B specific antibody. The increased signal of LC3B and the puncta structures were readily observed under confocal microscope after treatment with 2.5 μM and 5 μM tenovin-6 for 16 h (Figure 3D). Together, these results indicated that LC3B-II but not p53 activation was consistently increased after tenovin-6 treatment in DLBCL cell lines.

### Tenovin-6-mediated increase of LC3B-II is independent of SIRT1/2/3 and p53

It has been shown that tenovin-6 can directly inhibit the activities of SIRT1, 2 and 3, and activate p53 [17]. Because SIRT1, 2 and 3 as well as p53 also regulate the expression of LC3B-II [25–30], we investigated whether they might mediate LC3B-II increase in DLBCL cells following tenovin-6 treatment. The results showed that there was no correlation between the increase of LC3B-II level and p53 activation (Figure 3A). Furthermore, examination of time kinetic in OCI-Ly1 cells showed that tenovin-6 increased LC3B at as early as 0.5 h following treatment while it did not affect the level of p53 phosphorylation (Ser15) after up to 8 h of treatment (Figure 3C). Hence, p53 was not responsible for the increased LC3B-II level.

**Figure 4:**
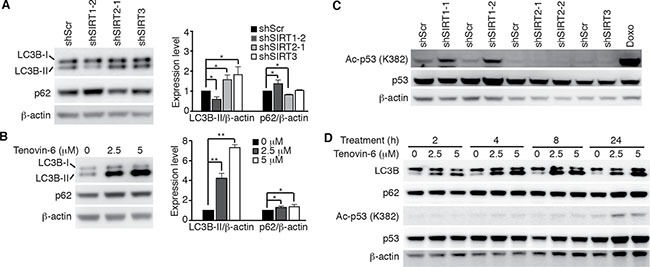
Tenovin-6-mediated increase of LC3B-II is SIRT1/2/3- and p53-independent (**A**) LC3B and SQSTM1/p62 (p62) expression levels in OCI-Ly1 cells following knockdown of SIRT1, 2 or 3 (left panel). The relative expression levels of LC3B-II and p62 from 3 independent experiments were quantified (right panel). *P < 0.05, **P < 0.01. (**B**) LC3B and SQSTM1/p62 (p62) expression levels in OCI-Ly1 cells after treatment with 2.5 μM or 5 μM tenovin-6 for 16 h (left panel). The relative expression levels of LC3B-II and p62 from 3 independent experiments were quantified (right panel). *P < 0.05, **P < 0.01. (**C**) The levels of acetylated p53 [Ac-p53 (K382)] and p53 in OCI-Ly1 cells following knockdown of SIRT1, 2 or 3. OCI-Ly1 cells treated with 2 μM doxorubicin (Doxo) for 7 h were used as a positive control. (**D**) The kinetics of acetylated p53 [Ac-p53 (K382)], p53, LC3B and SQSTM1/p62 (p62) in OCI-Ly1 cells after treatment with 2.5 μM or 5 μM tenovin-6.

We further examined LC3B expression and p53 acetylation in OCI-Ly1 cells following knockdown of SIRT1/2/3 or treatment with tenovin-6. LC3B-II was decreased by 40% following knockdown of SIRT1 and increased by 1.6- and 1.7-fold following knockdown of SIRT2 and SIRT3, respectively (Figure 4A) while it was increased by 4.2- and 7.3-fold following treatment with 2.5 μM and 5 μM tenovin-6, respectively (Figure 4B). p53 acetylation was slightly increased following knockdown of SIRT1 but remained unchanged following knockdown of SIRT2 or SIRT3 (Figure 4C). p53 acetylation did not increase until 24 h following treatment with 2.5 μM or 5 μM tenovin-6. However, LC3B-II started to increase at as early as 0.5 h following treatment with tenovin-6 (Figures 3C and 4D). These results indicated that the increase of LC3B-II after tenovin-6 treatment was independent of SIRT1/2/3 and p53. Our results confirmed those of previous studies in chronic lymphocytic leukemia [19] and soft tissue sarcoma cells [31], showing that the effect of tenovin-6 was p53-independent.

It has been shown that p27 is highly correlated with LC3B-II induction [32]. To test whether the effect of tenovin-6 on LC3B was through regulating p27 expression, we examined p27 expression at different time points after tenovin-6 treatment. The results showed that tenovin-6 treatment did not affect p27 expression (Figure 3C).

### Tenovin-6 induces apoptosis through the extrinsic cell-death pathway

**Figure 5:**
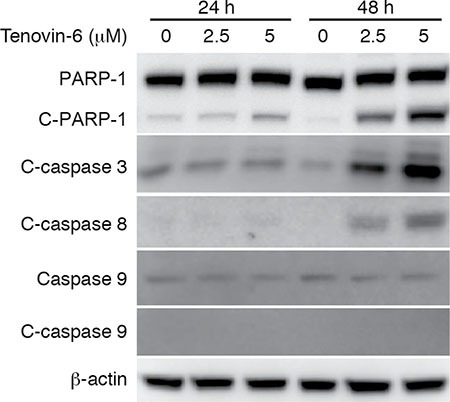
Tenovin-6 induces apoptosis through the extrinsic cell-death pathway The expression levels of apoptosis-related markers cleaved PARP-1 (C-PARP-1), cleaved caspase 3 (C-caspase 3), cleaved caspase 8 (C-caspase 8) and cleaved caspase 9 (C-caspase 9) in OCI-Ly1 cells following tenovin-6 treatment were examined by Western-blotting.

We observed significant apoptosis at 48 h after tenovin-6 treatment (Figure 1D and 1E). Apoptosis can be initiated by extrinsic or intrinsic pathway. Induction of apoptosis leads to activation of an initiator caspase: caspase-8 for the extrinsic pathway and caspase-9 for the intrinsic pathway [33]. To determine the apoptotic pathway triggered by tenovin-6, we examined apoptosis-related markers including cleaved PARP-1, cleaved caspase 3, cleaved caspase 8 and cleaved caspase 9 in OCI-Ly1 cells. Tenovin-6 treatment induced both cleaved PARP-1 and cleaved caspase 3 (Figure 5), which was consistent with the detection of increased apoptotic cells (Figure 1D and 1E). Furthermore, we observed cleavage of caspase 8 but not caspase 9 after tenovin-6 treatment (Figure 5), indicating that tenovin-6 likely activated the extrinsic but not the intrinsic cell-death pathway.

### Tenovin-6 increases LC3B-II by inhibiting the classical autophagy pathway

**Figure 6:**
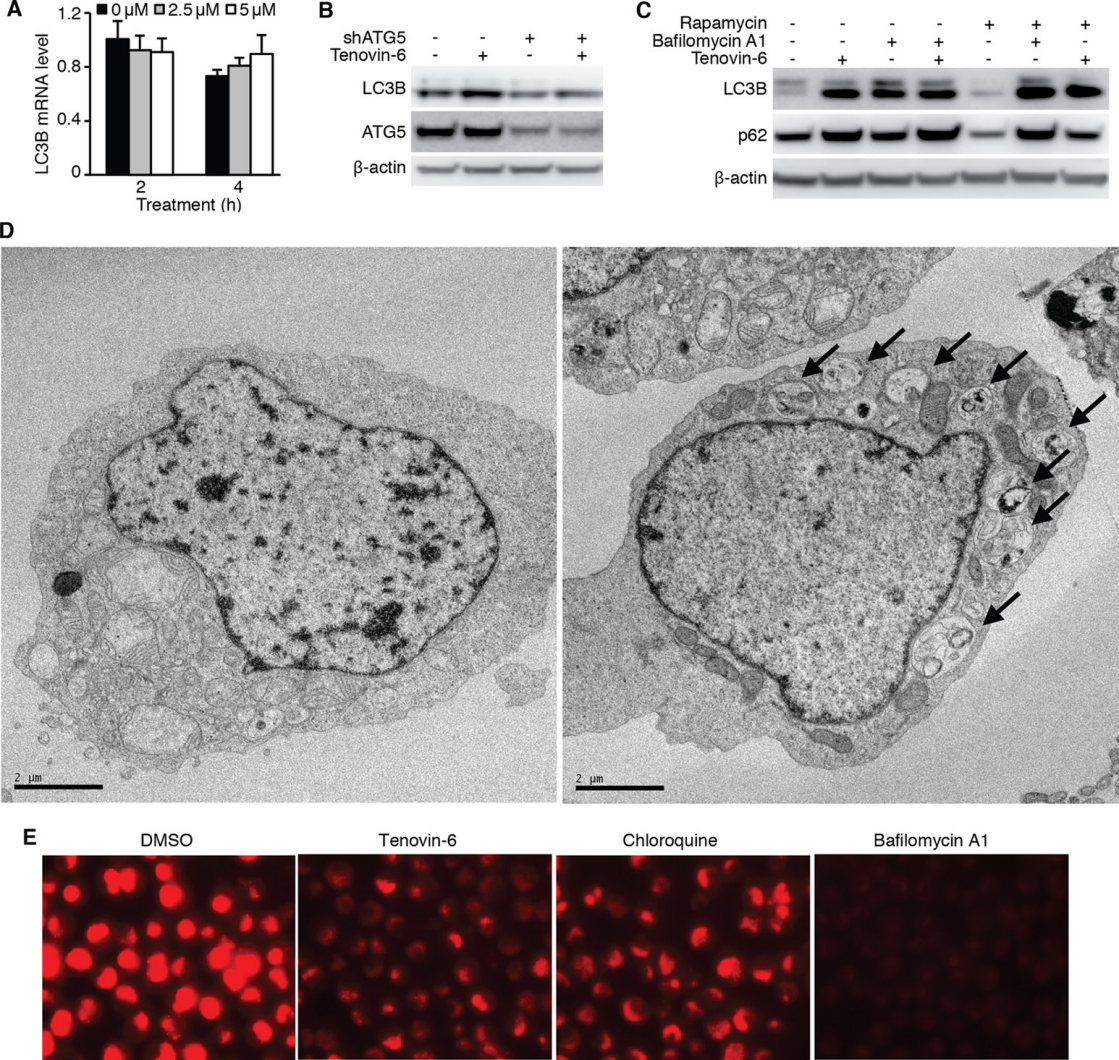
Tenovin-6 increases LC3B-II level by inhibiting the classical autophagy pathway (**A**) Relative LC3B mRNA analyzed by qPCR in OCI-Ly1 cells after treatment with 2.5 μM or 5 μM tenovin-6 for 2 h and 4 h. (**B**) LC3B-II was examined by Western-blotting in OCI-Ly1 cells with ATG5 knockdown following treatment with 5 μM tenovin-6 for 8 h. (**C**) LC3B-II and SQSTM1/p62 (p62) expression levels were examined by Western-blotting in OCI-Ly1 cells following combined treatment with 0.5 μM rapamycin, 50 nM bafilomycin A1 or 5 μM tenovin-6 for 16 h. (**D**) Representative transmission electron microscopy images of OCI-Ly1 cells following 5 μM tenovin-6 treatment for 16 h. (**E**) Lysotracker staining of OCI-Ly1 cells following treatment with 5 μM tenovin-6, 50 nM bafilomycin A1 or 100 μM chloroquine for at 1 h.

While LC3B-II is an autophagy marker, its level may be regulated at either transcriptional or post-translational level. Treatment of OCI-Ly1 cells with 2.5 μM or 5 μM tenovin-6 for 2 h or 4 h did not significantly alter the mRNA level of LC3B (Figure 6A) indicating that tenovin-6 did not transcriptionally regulate LC3B. ATG5 is essential for LC3-II processing in the autophagy pathway [34, 35]. To determine if the autophagy pathway was involved in regulating LC3B-II level, we first knocked down ATG5 in OCI-Ly1 cells and then treated the cells with tenovin-6. Knockdown of ATG5 significantly compromised the increase of LC3B-II caused by tenovin-6 treatment (Figure 6B), indicating that the classical autophagy pathway was required for tenovin-6-mediated increase of LC3B-II. Increased LC3B-II expression can be explained by either induction of autophagy or inhibition of autophagy flux. If autophagy is induced, SQSTM1/p62 should be degraded. On the other hand, if the autophagy flux is inhibited, SQSTM1/p62 should accumulate or remain unchanged. Since no SQSTM1/p62 degradation was observed following tenovin-6 treatment (Figures 3A, 3C, 4B and 4D), it suggested that tenovin-6 might inhibit the autophagic flux.

To confirm the inhibitory effect of tenovin-6 on autophagic flux, we treated OCI-Ly1 cells with tenovin-6 in the presence of a known autophagic flux inhibitor bafilomycin A1, and examined the effect on LC3B and SQSTM1/p62. If tenovin-6 is an inducer of the autophagic flux, the combined treatment of tenovin-6 with a saturated inhibitor like bafilomycin A1 should lead to an additive effect on LC3B expression. However, if tenovin-6 is an inhibitor of the autophagic flux, there should not be any additive effects because bafilomycin A1 should have completely blocked the autophagic flux. We did not observe any additive effect following combined treatment of 2.5 μM or 5 μM tenovin-6 with saturated bafilomycin A1, indicating that tenovin-6 was indeed an inhibitor of the autophagic flux (Figure 6C, Supplementary Figure 3). In contrast, combined treatment of rapamycin, an autophagy inducer, with bafilomycin A1 indeed showed an additive effect on the increased level of LC3B (Figure 6C, Supplementary Figure 3). Rapamycin alone induced SQSTM1/p62 degradation as expected [36]; however, it was inhibited by tenovin-6 or bafilomycin A1, further confirming tenovin-6 as an inhibitor of the autophagic flux.

To further confirm the inhibitory effect of tenovin-6 on autophagic flux, we examined the ultrastructure change of cultured OCI-Ly1 cells following tenovin-6 treatment by transmission electron microscopy analysis. We found that tenovin-6-treated cells had an increased number of double-membrane bound vacuoles within the cytoplasm. These vacuoles contained cellular debris and recognizable cytoplasmic organelles, confirming that they were accumulated autophagosomes (Figure 6D).

Because both tenovin-6 and bafilomycin A1 had similar inhibitory effects on the autophagic flux, we proposed that tenovin-6 might affect the function of lysosomes/autolysosomes leading to the inhibition of autophagy. Furthermore, our recent study has revealed that tenovin-6 inhibits the classical autophagy pathway by impairing lysosomal function [37]. LysoTracker is a cell-permeable pH-dependent dye that accumulates in acidic vesicles such as lysosomes or autolysosomes in live cells and emits a red fluorescence signal. LysoTracker staining has been commonly used to monitor the function of lysosome/autolysosome [38–41]. To determine whether tenovin-6 also inhibited the classical autophagy pathway by impairing lysosomal function in DLBCL cells, we treated OCI-Ly1 cells with tenovin-6, bafilomycin A1 or chloroquine, which is known to inhibit autophagy by elevating lysosomal pH, for 1 h, then stained the cells with LysoTracker. Tenovin-6, bafilomycin A1 or chloroquine significantly reduced the signal of LysoTracker staining (Figure 6E), suggesting that the function of lysosomes/autolysosomes were indeed affected by these inhibitors.

### Inhibition of the autophagy pathway impairs cell proliferation and survival of DLBCL cells

**Figure 7:**
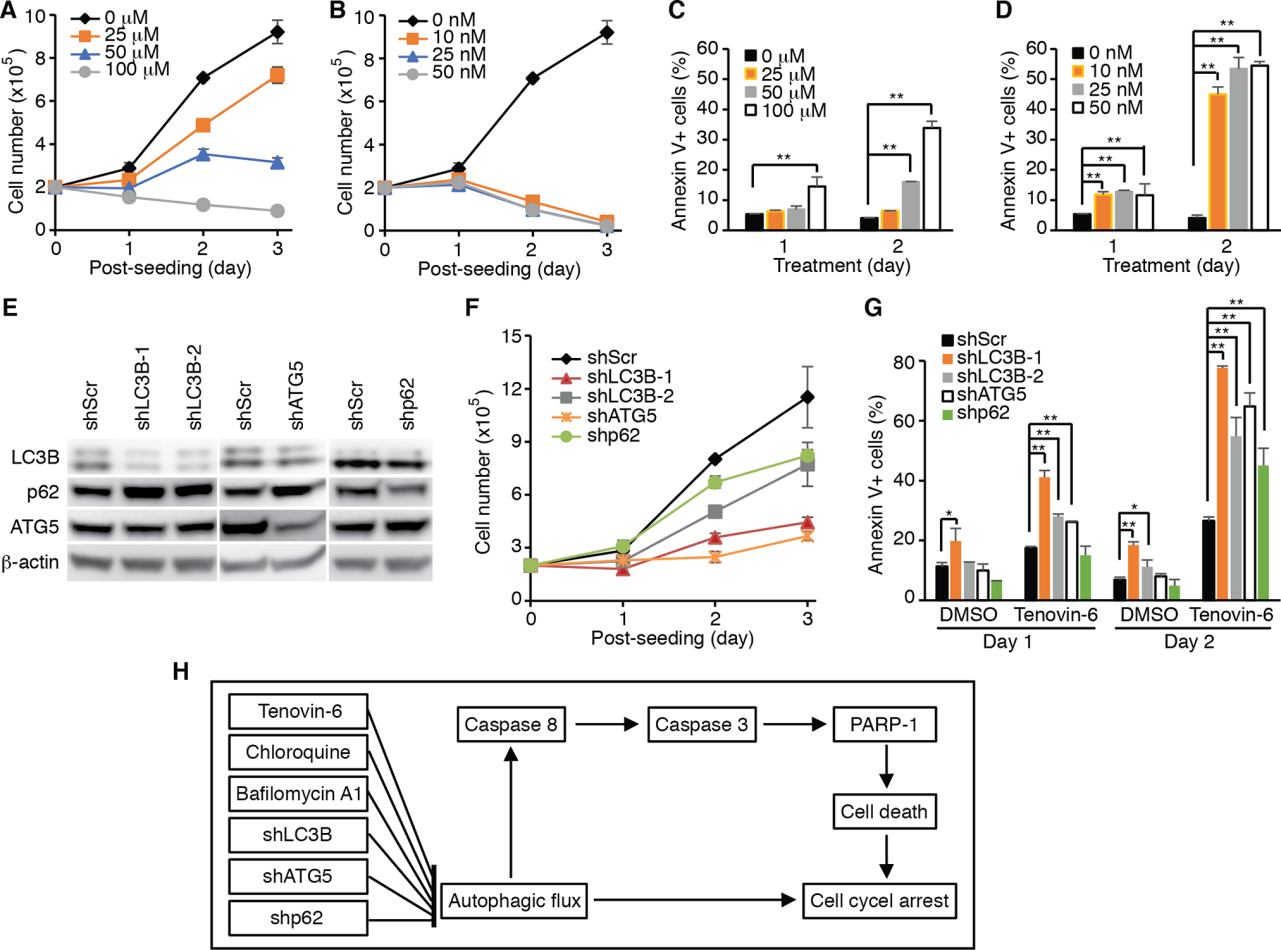
Inhibition of the autophagy pathway induces cell proliferation arrest and apoptosis in DLBCL cells (**A**) Growth curves of OCI-Ly1 cells after treatment with the indicated concentrations of chloroquine. (**B**) Growth curves of OCI-Ly1 cells after treatment with the indicated concentrations of bafilomycin A1. (**C**) The percentages of annexin V+ OCI-Ly1 cells after treatment with the indicated concentrations of chloroquine. *P < 0.05, **P < 0.01. (**D**) The percentages of annexin V+ OCI-Ly1 cells after treatment with the indicated concentrations of bafilomycin A1. *P < 0.05, **P < 0.01. (**E**) The expression levels of LC3B, ATG5 and SQSTM1/p62 (p62) in OCI-Ly1 cells were examined by Western-blotting following knockdown with their respective shRNAs. (**F**) Growth curves of OCI-Ly1 cells following knockdown of LC3B, ATG5 or SQSTM1/p62 (p62). (**G**) The percentages of annexin V+ OCI-Ly1 cells following knockdown of LC3B, ATG5 or SQSTM1/p62 (p62) with or without treatment with 5 μM tenovin-6. *P < 0.05, **P < 0.01. (**H**) Summary of the major findings in this study. Inhibition of autophagy either by using specific inhibitors or by specifically knocking down of key genes in the autophagy pathway leads to cell proliferation arrest and apoptosis in DLBCL cells.

The results so far suggested that the effect of tenovin-6 on DLBCL cells could be due to its inhibitory effect on autophagic flux rather than sirtuins. To test whether inhibition of the autophagic flux was sufficient to reduce cell proliferation and survival in DLBCL cells, we examined the inhibitory effects of known autophagy inhibitors bafilomycin A1 and chloroquine on cell proliferation and survival of OCI-Ly1 cells. Indeed, both bafilomycin A1 and chloroquine inhibited cell proliferation (Figure 7A and 7B) and induced apoptosis (Figure 7C and 7D) in a time- and dose-dependent manner. To further confirm these results, we performed knockdown of LC3B, ATG5 and SQSTM1/p62 genes (Figure 7E), which are critical for autophagosome formation at the early stage during the induction of the autophagy pathway [36]. Knockdown of LC3B, ATG5 or SQSTM1/p62 inhibited cell proliferation (Figure 7F) and induced apoptosis in OCI-Ly1 cells (Figure 7G). Importantly, treatment with tenovin-6 together with knockdown of LC3B, ATG5 or SQSTM1/p62 had an additive effect on the survival of cells (Figure 7G). These results indicated that approaches by combining inhibitors targeting different stages of the autophagy pathway might lead to better therapeutic outcomes for patients with DLBCL.

## DISCUSSION

The roles of sirtuins in cancer are complex and may either promote or suppress tumorigenesis depending on the cellular and molecular context [42]. In the present study, knockdown of SIRT1, 2 and 3 in DLBCL cells has no significant effect on cell proliferation and survival. However, the inhibitor of sirtuins tenovin-6 has a dramatic inhibitory effect on all 6 DLBCL cell lines examined, of which 4 belong to the ABC-type, a subset of incurable DLBCL. Tenovin-6 induced cell proliferation arrest and apoptosis in these DLBCL cells, strongly suggesting that tenovin-6 might have a different target other than sirtuins. This assumption is further supported by other evidences: 1) While in most published studies, tenovin-6 was used in the range of 1–5 μM, which is much lower than the IC50 values of 21, 10 and 67 μM for the purified human SIRT1, SIRT2 and SIRT3, respectively, it still demonstrated a strong effect on cell proliferation and survival [9–13, 15, 18, 19, 31, 43–47]; and 2) There was little or no correlation between acetylomes of cells with specific knockout of SIRT1 or SIRT2 and tenovin-6 treatment [48]. These results along with those of the current study suggest that precaution should be taken to interpret the data where tenovin-6 was used as an inhibitor of sirtuins. In the current study, we have revealed that tenovin-6 impairs autophagy, which is the underlying mechanism for its effect on DLBCL cells. In a recent study, we have examined the effect of tenovin-6 in several types of normal cells, mouse embryonic fibroblasts, a hepatocarcinoma cell line Huh7, a lung adenocarcinoma epithelial cell lines A549, a KSHV-transformed cell line KMM, and a DLBCL cell line OCI-Ly1 [37]. The results also showed that tenovin-6 inhibited the autophagy pathway. However, the effectiveness of using tenovin-6 as a potential therapeutic agent for DLBCL remains unknown. In the current study, we have, for the first time, shown tenovin-6 is effective against diverse cell lines of DLBCL of different genotypes, and hence can be used as a potential therapeutic agent for DLBCL.

Autophagy is an evolutionarily conserved cellular homeostatic process in response to starvation or other stress conditions in which proteins, organelles and intracellular pathogens are engulfed into autophagosomes and eventually delivered to lysosomes for degradation. The autophagy pathways are involved with a large number of protein complexes. The ATG1 complex, the ATG2-ATG18 complex, the class III phosphatidylinositol 3-kinase (PI3K) complex, the transmembrane protein mATG9 complex (and its vesicular trafficking along with ATG16L1), and the ATG8 and ATG12 ubiquitin-like conjugation complexes are critical for autophagosome formation. Cytoskeletons, RAB GTPases, HOPS (homotypic fusion and vacuolar protein sorting) complex, and SNAREs are important for autophagosome-lysosome fusion. The blockage of autophagy can disrupt its protective effect against starvation and other stresses, and cause cell cycle arrest and cell death [49].

The role of autophagy in cancer has been extensively studied and the results have been reviewed [50–54]. It is now well established that autophagy functions as a suppressor in tumor initiation at the early stage of tumor progression while it promotes tumor progression at the advanced stages of tumor development. The established tumors appear to utilize autophagy for surviving the stresses in the tumor microenvironment including low oxygen, low nutrients, excess toxicities and attack of immune cells [50–54]. Autophagy is activated in cancer cells undergoing treatment with different chemotherapeutic drugs such as cisplatin, etoposide, 5-fluorouracil and doxorubicin, and genetic knockdown of autophagy genes or pharmacological inhibition of autophagy with the use of chloroquine, 3-methyladenine or bafilomycin A1 sensitizes cancer cells to chemotherapeutic drugs [55–60]. These studies suggest that suppression of the autophagic pathway can be a novel strategy for anticancer therapy. Currently, various clinical trials are investigating the effects of combining autophagy inhibitors with cytotoxic chemotherapies and targeted agents in solid and hematological cancers. The early results of some of these clinical studies indicate that autophagy inhibition in combination with anticancer therapies seem to be safe and can augment the efficacies of various anticancer therapies in melanoma, glioblastoma and multiple myeloma [50, 53]. In addition to the existing autophagy inhibitors such as hydroxychloroquine and chloroquine, which are approved for treatment of malaria by US FDA, identification of novel and highly effective autophagy inhibitors is obviously needed. Tenovin-6 seems to be a good candidate for use in combined chemotherapies: First, this study has demonstrated tenovin-6 as a potent autophagy inhibitor; Second, tenovin-6 seems to be safe since no obvious adverse effect has been reported so far in multiple studies in the mice [9, 11, 12, 17, 31]; and Third, tenovin-6 alone has shown a promising anti-neoplastic effect *in vitro* or *in vivo* on various hematopoietic and solid malignancies [9–13, 15, 18, 19, 31, 43–47].

The information on the role of autophagy in DLBCL cells is very limited so far. Only one specific study has been published, showing that proteasome inhibitor bortezomib-induced autophagy conferred relative drug resistance to DLBCL cells by eliminating IκBα, and the inhibition of both autophagy and proteasome had demonstrated a great potential for killing apoptosis-resistant DLBCL cells [61]. In a relevant clinical trial in dogs with non-Hodgkin's lymphoma, the combined treatment of autophagy inhibitor hydroxychloroquine (12.5 mg/kg/d) with a lower dose of doxorubicin (25 mg/m^2^) has resulted in clinical benefit with superior overall response rate (93.3%) and comparable median progression-free interval (5 months) compared to standard single agent doxorubicin (30 mg/m^2^) treatment [62]. In the current study, we have found that inhibition of autophagy, either by using inhibitors or by specifically knocking down key genes of the autophagy pathway, can induce cell proliferation arrest and apoptosis in DLBCL cells (Figure 7H). Our results have illustrated an essential protective role of autophagy in DLBCL cells and strongly suggested that targeting autophagy may represent a novel strategy for overcoming the chemoresistance, which is the major current challenge for treating DLBCL.

## MATERIALS AND METHODS

### Reagents and antibodies

The following reagents were used in this study: tenovin-6 (BSCC-37, Agave Pharm, Seattle, WA), chloroquine diphosphate (C6628, Sigma, St. Louis, MO), bafilomycin A1 (B1793, Sigma), rapamycin (R8781, Sigma), and LysoTracker Red DND-99 (L7528, Thermo Fisher Scientific, Waltham, MA).

The antibodies used were: LC3B (CTB-LC3-1-50, Cosmo Bio, Carlsbad, CA), LC3A (ab62720, Abcam, Cambridge, MA), SQSTM1/p62 (PM045, MBL, New York, NY), ATG5 (#2630, Cell Signaling Technology, Danvers, MA), β-actin (sc-8432, Santa Cruz, Dallas, TX), β-tubulin (T8453, Sigma), p53 (DO-1, sc-126, Santa Cruz), acetyl-p53 (K382) (#2524, Cell Signaling Technology), Phospho-p53 (S15) (#9284, Cell Signaling Technology), Bax (#2772, Cell Signaling Technology), Puma (#4976, Cell Signaling Technology), p21 (#556430, BD Biosciences, Franklin Lakes, NJ), PARP-1 (#9532, Cell Signaling Technology), c-caspase 3 (#9664, Cell Signaling Technology), caspase 8 (#9746, Cell Signaling Technology), c-caspase 9 (SC-7885, Santa Cruz), SIRT1 (#8469, Cell Signaling Technology), SIRT2 (#12672, Cell Signaling Technology), SIRT3 (#2627, Cell Signaling Technology), SIRT4 (NB100-1406, Novus, St Charles, MO), SIRT5 (#8782, Cell Signaling Technology), SIRT6 (#2590, Cell Signaling Technology), and SIRT7 (#5360, Cell Signaling Technology).

### Cell culture

HEK 293T cells were cultured in Dulbecco's Modified Eagle's Medium. RIVA, U2932, HBL1, SU-DHL4, SU-DHL5, SU-DHL6 and SU-DHL10 cells were cultured in RPMI-1640 media. OCI-Ly1, OCI-Ly3 and OCI-Ly10 were cultured in Iscove's Modified Dulbecco's Medium. All media was supplemented with 100 μg/mL penicillin, 100 μg/mL streptomycin and 10% fetal bovine serum (#26140-079, Thermo Fisher Scientific) except that 20% fetal bovine serum was used for OCI-Ly3 and OCI-Ly10. All cells were maintained in a 5% CO2 atmosphere at 37°C.

### Lentiviral shRNA knockdown

The ATG5 shRNA (TRCN0000150940, 5′-GCAGAACCATACTATTTGCTT-3′), LC3B shRNA (#1: TRCN0000154060, 5′-CGCTTACAGCTCAATGC TAAT-3′ and #2: TRCN0000155417, 5′-CCTGACCAT GTCAACATGAGT-3′), and SQSTM1/p62 shRNA (TRCN0000007236, 5′-CCGAATCTACATTAAAGAGA A-3′) were purchased from Sigma. The SIRT1 shRNA (#1: 5′-GAAGTGCCTCAGATATTAA-3′ and #2: 5′-GTTGACCTCCTCATTGTTA-3′), SIRT2 shRNA (#1: 5′-GCTTATTGGAGACAAATTA-3′ and #2: 5′-GAAACATCCGGAACCCTTC-3′) and SIRT3 shRNA (5′-GGAGTGGCCTGTACAGCAA-3′) were constructed as previously described [9]. A scrambled shRNA (5′-TTGTACTACACAAAAGTACTG-3′) was constructed as a control. The lentivirus was generated and concentrated as previously described [9]. Cells were transduced with the lentivirus particles by spinning infection at 1,800 rpm for 60 min in the presence of 8 μg/ml polybrene at MOI 12. At 48 h post-transduction, cells were selected with 2.5 μg/ml puromycin for 3 days, cultured for another 3 days and used for the experiments.

### BrdU-PI staining and annexin V staining

BrdU-PI staining was performed as previously described [63]. Briefly, 20 μM BrdU (B5002, Sigma) was added into the culture media for 4 h, fixed and stained with a Pacific Blue monoclonal antibody to BrdU (B35129, Thermo Fisher Scientific) and propidium iodide (P4864, Sigma) for DNA. PE-Cyanine 7-conjugated anti-annexin V antibody (25-8103-74, eBioscience, San Diego) was used to detect apoptosis following the instructions of the manufacturer. Samples were run in a FACSCanto System (BD Biosciences) and analyzed with FlowJo (Treestar, Ashland, OR).

### Quantitative real-time PCR (qPCR)

qPCR was performed as previously described [64]. Briefly, total RNA was isolated with TRI reagent (T9424, Sigma). Reverse transcription was performed with total RNA using Maxima H Minus First Strand cDNA Synthesis Kit (K1652, Thermo Fisher Scientific,). qPCR analysis was performed on Eppendorf Real Plex using KAPA SYBR FAST qPCR Kits (KK4602, Kapa Biosystems, Wilmington, MA). The relative expression levels of the targeted genes were normalized to the expression of internal control 18S rRNA gene. All reactions were run in triplicate. Sequences for LC3B primers were: human LC3B_F: 5′-CCGCACCTTCGAACAAAGAGT-3′ and LC3B_R: 5′-CACCCTTGTATCGTTCTATTATCACC-3′. Sequences for 18S rRNA primers were: human 18S rRNA_F: 5′-ATCAACTTTCGATGGTAGTCG-3′ and 18S rRNA_R: 5′-TCCTTGGATGTGGTAGCCG-3′.

### Immunofluorescence assay

OCI-Ly1 cells were treated as indicated and cytospun onto glass slides at 800 rpm for 3 min. After fixation with 2% paraformaldehyde for 20 min at room temperature, the cells were washed and blocked with 3% BSA in PBS for 30 min at 37°C. The cells were then incubated with an anti-LC3B antibody (CTB-LC3-1-50, Cosmo Bio) at a 1:500 dilution for 1 h at 37°C. After incubation for 1 h at 37 °C with a secondary antibody (A-11001, ThermoFisher Scientific), samples were counterstained with 0.5 μg/mL 4-,6-diamidino-2-phenylindole (DAPI) in PBS for 5 min, and the slides were mounted in FluorSave Reagent (#345789, Calbiochem, Billerica, MA). Samples were observed with a laser-scanning confocal microscopy (Nikon Eclipse C1).

### Electron microscopy

The cell pellets were fixed with 2% glutaraldehyte in 0.1M cacodylate buffer [Na(CH_3_)_2_AsO_2_·3H_2_O], pH7.2, at 4°C, overnight. The cell pellets were washed three times with 0.1M cacodylate buffer, pH7.2, post-fixed with 1% OsO_4_ in 0.1M cacodylate buffer for 30 min and washed three times with 0.1M cacodylate buffer. The samples sequentially dehydrated once with 60%, 70%, 80% and 95% ethanol, twice with 100% absolute ethanol, and twice with propylene oxide were left in propylene oxide/Eponate (1:1) overnight at room temperature. The vials were sealed. The next day the vials were left open for 2–3 h to allow the propylene oxide to evaporate. The samples were infiltrated with 100% Eponate and polymerized at ~64°C for 48 h. Ultra-thin sections (~70 nm thick) were cut using a Leica Ultra cut UCT ultramicrotome with a diamond knife and picked up on 200 mesh copper EM grids. Grids were stained with 2% uranyl acetate for 10 min followed by Reynold's lead citrate staining for 1 min. The grids were observed with an FEI Tecnai 12 transmission electron microscope equipped with a Gatan Ultrascan 2K CCD camera (FEI, Hillsboro, OR).

### LysoTracker staining

Cells were stained with 50 nM LysoTracker Red DND-99 (ThermoFisher Scientific) by directly adding this dye to the culture media, incubated for 30 min in a 5% CO2 atmosphere at 37°C, and observed with a Nikon Eclipse microscopy (Tokyo, Japan).

### Western-blotting

Western-blotting was performed as previously described with minor modifications [64]. Briefly, cells were lysed in RIPA buffer supplemented with 1% SDS and proteinase inhibitor cocktail (1:100) (P8340, Sigma). Protein lysate of 10 μg for each sample was resolved by SDS-PAGE and transferred onto a PVDF membrane. After blocking with 5% skim milk, the membrane was probed with primary and then secondary antibodies. The signal was developed with the Luminiata Crescendo Western HRP substrate (WBLUR0500, EMD Millipore, Billerica, MA) and visualized with a Fujifilm LAS-3000 imaging system (Redondo Beach, CA).

### Statistical analysis

Two-tailed *t-test* was performed, and *P* < 0.05 (*) and *P* < 0.01 (**) were considered statistically significant.

## SUPPLEMENTARY MATERIALS FIGURES


