# Supplementary material for: Tenovin-6 inhibits proliferation and survival of diffuse large B-cell lymphoma cells by blocking autophagy
Source: Oncotarget. 2017 Jan 19;8(9):14912–24. doi: 10.18632/oncotarget.14741 (PMC5362454; doi:10.18632/oncotarget.14741)
Supplement: Supplementary file 1 [file oncotarget-08-14912-s001.pdf]

Tenovin-6 inhibits proliferation and survival of diffuse large B-cell lymphoma cells by blocking autophagy

Supplementary Materials

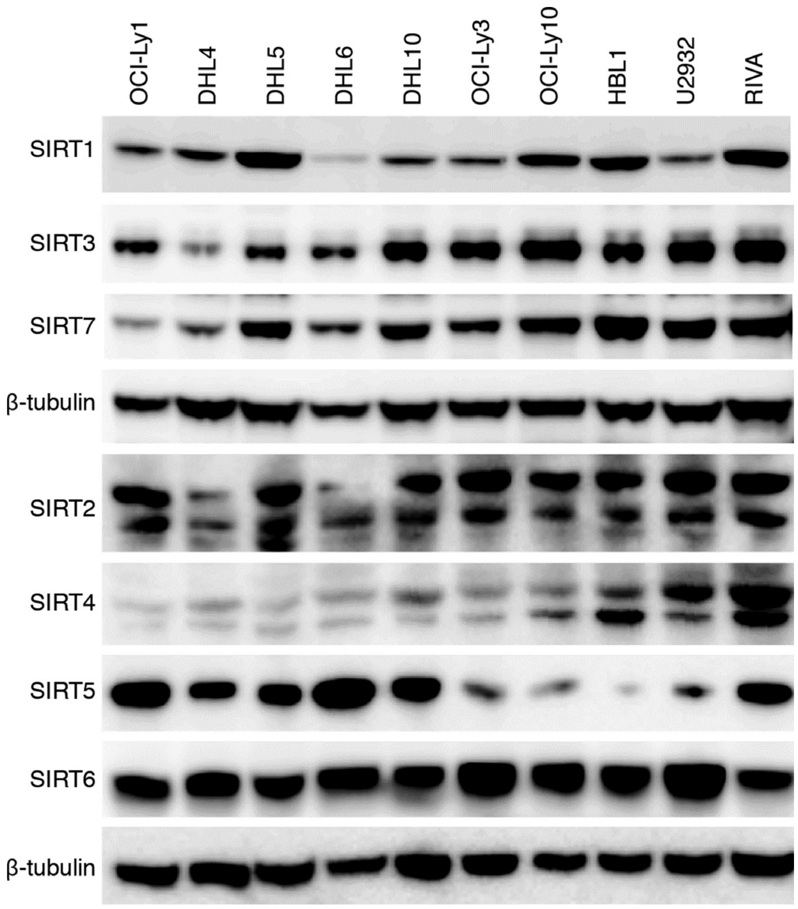

Supplementary Figure 1: The expression levels of sirtuins were examined by Western-blotting in diffuse large B-cell lymphoma cell lines.

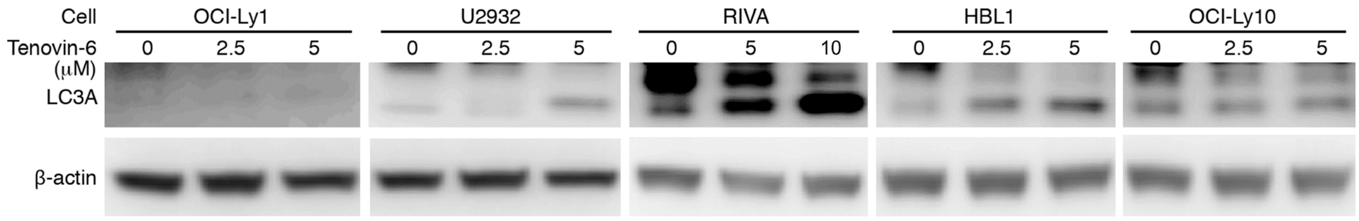

Supplementary Figure 2: The expression level of LC3A was examined by Western-blotting in diffuse large B-cell lymphoma cell lines following treatment with the indicated of concentrations of tenovin-6 for 24 h.

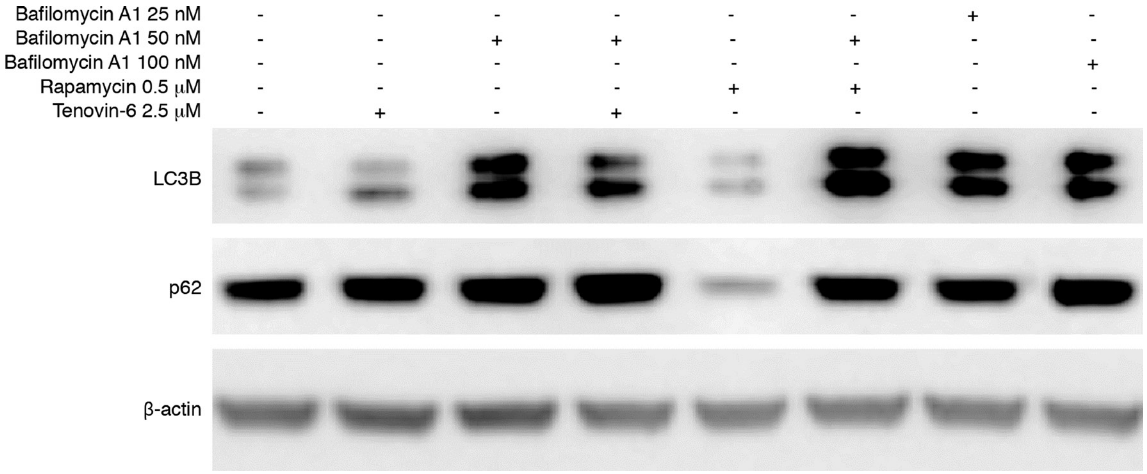

Supplementary Figure 3: The expression levels of LC3B-II and p62 were examined by Western-blotting in OCI-LY1 cells following combined treatments with the indicated concentrations of rapamycin, bafilomycin A1 and tenovin-6 for 16 h.
